# Synergy in Efficacy of Fungal Entomopathogens and Permethrin against West African Insecticide-Resistant *Anopheles gambiae* Mosquitoes

**DOI:** 10.1371/journal.pone.0012081

**Published:** 2010-08-11

**Authors:** Marit Farenhorst, Bart G. J. Knols, Matthew B. Thomas, Annabel F. V. Howard, Willem Takken, Mark Rowland, Raphael N’Guessan

**Affiliations:** 1 Laboratory of Entomology, Wageningen University and Research Center, Wageningen, The Netherlands; 2 Division of Infectious Diseases, Tropical Medicine & AIDS, Academic Medical Center, Amsterdam, The Netherlands; 3 Department of Entomology, Center for Infectious Disease Dynamics, Pennsylvania State University, University Park, Pennsylvania, United States of America; 4 Malaria Center, London School of Hygiene and Tropical Medicine, London, United Kingdom; 5 Centre de Recherches Entomologiques de Cotonou, Laboratoire Nationale Ministère de la Santé, Cotonou, Benin; University of California Los Angeles, United States of America

## Abstract

**Background:**

Increasing incidences of insecticide resistance in malaria vectors are threatening the sustainable use of contemporary chemical vector control measures. Fungal entomopathogens provide a possible additional tool for the control of insecticide-resistant malaria mosquitoes. This study investigated the compatibility of the pyrethroid insecticide permethrin and two mosquito-pathogenic fungi, *Beauveria bassiana* and *Metarhizium anisopliae*, against a laboratory colony and field population of West African insecticide-resistant *Anopheles gambiae s.s*. mosquitoes.

**Methodology/Findings:**

A range of fungus-insecticide combinations was used to test effects of timing and sequence of exposure. Both the laboratory-reared and field-collected mosquitoes were highly resistant to permethrin but susceptible to *B. bassiana* and *M. anisoplia*e infection, inducing 100% mortality within nine days. Combinations of insecticide and fungus showed synergistic effects on mosquito survival. Fungal infection increased permethrin-induced mortality rates in wild *An. gambiae s.s*. mosquitoes and reciprocally, exposure to permethrin increased subsequent fungal-induced mortality rates in both colonies. Simultaneous co-exposure induced the highest mortality; up to 70.3±2% for a combined *Beauveria* and permethrin exposure within a time range of one gonotrophic cycle (4 days).

**Conclusions/Significance:**

Combining fungi and permethrin induced a higher impact on mosquito survival than the use of these control agents alone. The observed synergism in efficacy shows the potential for integrated fungus-insecticide control measures to dramatically reduce malaria transmission and enable control at more moderate levels of coverage even in areas where insecticide resistance has rendered pyrethroids essentially ineffective.

## Introduction

Malaria continues to have a major impact on health and economic development in Africa. Amongst numerous factors contributing to this problem, the increasing spread of insecticide resistance in the primary mosquito vector species is a major threat to contemporary malaria control efforts, which rely heavily on insecticide-based interventions such as Long-Lasting Insecticide Nets (LLINs) and Indoor Residual Spraying (IRS) [1]–[6].

Pyrethroids and dichlorodiphenyltrichloroethane (DDT) are the most widely used insecticides against malaria vectors, and act on the insect's central nervous system by blocking neuronal activity and causing rapid paralysis and death. Resistance to these insecticides in key vector species, such as *Anopheles gambiae*, can be conferred by a point-mutation in the target site, the sodium channel gene, which is known as knock-down resistance (*kdr*) [7], [8]. Because pyrethroids and DDT have a similar mode of action, this single target-site modification confers cross-resistance to both insecticide classes. Additionally, resistance can be the result of enhanced metabolic degradation of the insecticide by specific enzymes. Elevated levels of monooxygenases, esterases or glutathione S-transferases have been shown to confer resistance to insecticides in malaria vectors [5], [9]. Moreover, it is not uncommon for mosquitoes to exhibit a combination of resistance mechanisms, with both target-site and metabolic resistance determining the overall resistance phenotype [2], [10].

Given the importance of insecticide-based interventions for malaria control, development of strategies to avert the selection of resistance or to control resistant mosquitoes is paramount. Potential approaches include deployment of different insecticides in rotations or mosaics and development of novel insecticide classes [11], [12]. However, with problems of cross-resistance amongst existing chemicals and no new class of public health insecticide having reached the market for more than three decades [3], practical options for simple chemical-based approaches are limited. In this regard, there is increasing emphasis on the development of novel integrated vector control strategies.

A growing body of empirical and theoretical studies suggests a potential role for a new class of bio-insecticides based on insect-pathogenic fungi. Several laboratory studies have demonstrated the potential of the entomopathogenic fungi *Metarhizium anisopliae* and *Beauveria bassiana* to infect and kill *Anopheles*, *Aedes* and *Culex* mosquitoes [13]–[23]. Spores (conidia) of these two hyphomycetous fungi can attach to the insect upon contact, whereupon they germinate, penetrate the cuticle, proliferate inside the mosquito body and eventually cause death [24]. The infection process takes several days, usually between 3 and 14 days, with the overall time to death depending mostly on fungal dose and virulence of the isolate [17], [20], [23]. This mode of infection lends itself to a range of delivery systems. Several application techniques that use either dry or formulated spores on mosquito resting surfaces have been shown to infect and kill the majority of exposed mosquitoes within 7–10 days [16], [17], [19], [25]. Prior to death, fungal infection can also lead to reduced blood-feeding frequency and reproductive fitness [22] and can impact on the development of malaria parasites within the mosquito [21]. Other studies demonstrate low risk of spore applications to human health and the environment [26]–[28].

With respect to insecticide resistance, an important finding is that candidate fungal pathogens appear equally effective in infecting and killing metabolically resistant anophelines as their susceptible counterparts [18], [29]. A recent study showed that fungal impact was higher in a pyrethroid-resistant (*kdr*) colony of *An gambiae s.s*. than in an insecticide-susceptible colony [30]. Moreover, infection with *Metarhizium* or *Beauveria* increased permethrin and DDT sensitivity in highly resistant laboratory-reared *Anopheles* mosquitoes originating from Southern and East Africa, which was suggested to have been caused by a reallocation of insecticide-detoxifying enzymes toward fungal toxins [18]. These findings suggest potential for novel integrated vector management strategies that combine conventional and bio-insecticidal tools. Further support for this idea is provided by a recent theoretical study, which demonstrated that control strategies using both fungi and insecticide treated bednets could have greater impact on malaria transmission than control measures based on either intervention alone [31]. Such approaches could be of particular use in countries like Benin, where high levels of pyrethroid resistance are already threatening the impact of conventional vector control tools [4], [32].

Pyrethroid-treated LLINs are currently the primary malaria prevention intervention in Africa and, realistically, fungal-based vector control measures will far more likely be implemented in combination with LLINs than used as a substitute. The current study, therefore, explored the interactions between pyrethroids and fungi. Combinations of *M. anisopliae*, *B. bassiana* and permethrin were tested against laboratory-reared and field-collected West African *An. gambiae s.s.* mosquitoes, which were highly resistant to pyrethroids and DDT through the expression of the *kdr* gene. For optimum design of integrated fungus-insecticide field delivery formats, effects of timing and sequence of exposure were tested. Implementation of LLINs combined with indoor residual fungal treatments may result in mosquito contact to both products during a single feeding episode. Alternatively, mosquitoes may contact the fungus and insecticide in subsequent feeding cycles, for example when LLINs are combined with fungus-impregnated resting sites (such as clay pots [16], cotton ceiling cloths [19] or outdoor odour-baited stations [25]). Experiments, therefore, included simultaneous and sequential exposure combinations of fungus and permethrin to test effects on mosquito survival.

## Materials and Methods

### Fungus

Spores of *Metarhizium anisopliae var. anisopliae*, isolate ICIPE-30 (courtesy Dr. N. Maniania, ICIPE, Kenya), and *Beauveria bassiana*, isolate IMI 391510, were produced by solid state fermentation using glucose-impregnated hemp as a substrate (courtesy F. van Breukelen and M. Jumbe, Wageningen University, The Netherlands). After a standard growth period of 10 days, spores were dried at ambient temperature until moisture content was <5% and were subsequently harvested from the growth medium through sieving. Dry spores were stored in 50 ml sealed plastic tubes in the dark at 4°C until use.

For mosquito bioassays, spores were formulated in the synthetic isoparaffinic hydrocarbon solvent Shellsol T (Shellsol T®, Shell, The Netherlands) [17]. Formulations were mixed by vortexing and sonication for 10 seconds at 1000 Hz with a Branson probe sonicator (Branson B12, G. Heinemann, Germany). Spore concentration was determined with a Bürker-Türk haemocyte counter (W. Schreck, Hofheim/TS) under a light microscope (400×magnification) to quantify the number of spores per ml. The viability of fungal spores was assessed by scoring the proportion of germinated spores on Sabouraud dextrose agar with 0.001% Benomyl added (counting ≥300 spores/agar plate) after incubation at 27°C for 22–26 hours, using a light microscope (400×).

### Mosquitoes

The laboratory colony (named VKPer) consisted of *An. gambiae s.s*. (S-form) mosquitoes originating from the Kou Valley in Burkina Faso that were homozygously fixed for the *kdr* gene [7] and maintained in the insectary of the CREC institute in Cotonou, Benin. Eggs of this colony were shipped to the Laboratory of Entomology, Wageningen University, The Netherlands and a colony was started there. Larvae were reared in plastic trays filled with tap water and fed on Tetramin Flakes® fish food (Tetra, Melle, Germany). Adults were fed *ad libitum* on a 6% glucose/water (w/v) solution and maintained in 30×30×30 cm cages inside climate-controlled rooms (27±1°C, 80±10%). Exposure experiments on VKPer mosquitoes were also performed in these climate rooms, using 3–5 day old females.

The field colony consisted of adult mosquitoes reared from field-collected larvae and pupae obtained from breeding sites near Ladji, Benin (6°23′23N, 2°25′56E) in April 2009. Previous studies showed that in this location the anopheline population consists of resistant (*kdr*) *An. gambiae s.s.* (M-form) mosquitoes [2]. *Anopheles gambiae* larvae were separated from the field samples and reared in large, round plastic trays in the insectary of the CREC. Larvae were reared in plastic trays filled with tap water and fed on locally purchased cat food. Adults were maintained in the CREC insectary (26±1°C, >80% RH) and fed *ad libitum* on honey-water mixtures. Bioassays on the field-collected mosquitoes were performed in the CREC laboratory, in which temperature was maintained at approximately 20±2°C during the day, and at 26±1°C during observation periods (6 pm–8 am) with humidity >80% RH.

### Baseline fungal bioassays

The effect of fungal infection on mosquito survival was tested using a standardized exposure bioassay involving fungus-coated papers [17]. The K-Hand Coater (RK Print Coat Instruments Ltd., UK) was used to coat exposure papers with *B. bassiana* or *M. anisopliae* spores that were suspended in Shellsol T. On each A4 size paper, 0.9 ml of a 4.2×10^9^ spore/ml suspension was pipetted at the top of the 25×15 cm application surface, and coated manually onto the paper with a 0.31 mm wired K-bar (K bars ®, RK Print Coat Instruments Ltd., United Kingdom) that produced a 24 µm film deposit [17]. Control papers were treated with 0.9 ml Shellsol. The effective spore end-concentration comprised 10^11^ spores/m^2^ and was optimized to cause high levels of infection whilst not causing too rapid mortality, in order to monitor possible interaction effects over time. This exposure dose was also used for fungus-insecticide exposure experiments.

Papers were left to dry overnight in a climate-controlled room (27±1°C, 70±10% RH) before being placed inside a PVC-tube of 15 cm long and 8 cm diameter. Papers covered the entire inside surface of the tube and were fixed with two paperclips. Each tube was sealed with plastic microwave foil on either end, on which mosquitoes did not tend to rest. For each replicate, approximately 30 female mosquitoes were exposed to the papers for 1 hr and subsequently transferred to clean holding buckets via free flight [17]. Daily mosquito mortality was recorded and dead mosquitoes were removed from each bucket and checked for fungal infection by dipping cadavers in 70% ethanol to remove external microbiota (which does not affect the internally growing fungus) and incubating them on moist filter paper in sealed Petri dishes at 27±1°C. After 3–5 days mosquito cadavers were examined for fungal sporulation, i.e., emerging hyphae, using a dissection microscope. Because low infection doses and external factors, such as microbiota and temperature, can affect fungal growth [33], hyphal growth from cadavers is not a direct indicator of fungal infection and was only used as a positive control observation. Tests comprised four treated and control replicates for the VKPer strain and three replicates for the field-collected mosquitoes, set up on separate days using different mosquito batches.

### Fungus-insecticide combination assays

The effect of fungus and insecticide combinations on mosquito mortality was tested with a range of exposures and sequences, designed to mimic the sequence and timing of insecticide and fungal exposures that might occur under different scenarios of deployment in the field. Table 1 provides an overview of the various treatment combinations, and group numbers indicated in this table are used subsequently to describe treatments in the results. Mosquitoes were exposed to insecticide, fungal spores, or both, using standard WHO bioassay procedures [34] as described below. A three day interval was chosen between the two exposure rounds to represent the average duration of the gonotrophic cycle of *An. gambiae* and hence, the period between consecutive blood meals. This time-point was used in previous assessments on fungal impact on insecticide sensitivity [18] and corresponded to the start of fungal proliferation and the first noticeable impact on mosquito survival and allowed for measurements on fungal impact whilst not losing too many insects through death.

**Table 1:** Overview of insecticide and fungus exposure treatments.

		Exposure 1	Exposure 2
	Group	(Day 0)	(Day 3)
Controls	1	Control	Control
	2	Control	Perm
	3	Perm	Control
	4	Perm	Perm
*Beauveria*	5	Bb	Control
	6	Bb	Perm
	7	Bb + Perm	Control
	8	Bb + Perm	Perm
*Metarhizium*	9	Ma	Control
	10	Ma	Perm
	11	Ma + Perm	Control
	12	Ma + Perm	Perm

Mosquito cohorts were exposed on Day 0 for 1 hr to control papers (Control) or papers treated with permethrin (Perm), *B. bassiana* (Bb) or *M. anisopliae* (Ma). Bb+Perm and Ma+Perm represent groups exposed first to fungus and immediately after to insecticide. Survivors were subsequently exposed 1 hr to control or permethrin papers on Day 3.

#### Exposure 1

In the first exposure round, cohorts of ca. 28 females were transferred to WHO bioassay tubes with an aspirator and exposed for 1 hour to the treatments indicated in Table 1. Control groups were exposed to untreated papers. Insecticide exposures used papers treated with 0.75% permethrin from one single WHO production batch (Vector Control Reference Unit, Universiti Sains Malaysia, Penang, Malaysia). For fungal exposures, mosquitoes were exposed to paper coated the previous day with *B. bassiana* or *M. anisopliae* (10^11^ spores/m^2^). Effects of co-exposure were tested by exposing mosquitoes first for 1 hr to fungus-impregnated papers and immediately afterwards for 1 hr to permethrin papers. After exposure, mosquitoes were transferred to holding buckets via free flight and mortality was measured 24 hrs and 3 days after exposure.

#### Exposure 2

Three days after the first exposure, surviving mosquitoes were once more transferred from the holding buckets to WHO bioassay tubes and exposed either to permethrin papers or to control papers as indicated in Table 1. Exposures were performed as described above, for 1 hr, after which mosquitoes were transferred back to holding buckets. Mortality was scored after 24 hours (Day 4) and 3 days after the second exposure round (Day 7). Dead mosquitoes were removed checked for fungal infection, i.e. sporulation as described above. Mosquitoes that were still alive on Day 7 were removed from the buckets with an aspirator and killed by drowning in 70% alcohol before examining for fungal infection.

Permethrin-impregnated papers were re-used for a maximum period of two weeks and checked for efficacy (after use in exposure assays) by exposing insecticide-susceptible mosquitoes to the papers. In Wageningen, two groups of 25 female *An. gambiae s.s.* of the Suakoko strain were exposed (originating from Liberia, reared in Wageningen). In Cotonou, two groups of 25 female *An. gambiae s.s.* of the Kisumu strain were exposed (originating from Kenya, reared in Cotonou). Experimental data were only used if the insecticide papers induced 100% mortality in these susceptible strains.

### Data analysis

Differences in mosquito survival between fungus-infected and control groups were analyzed using Cox Regression with SPSS 16.0 software [35]. For both mosquito strains, survival curves of *Beauveria*- or *Metarhizium*-infected were compared to control mosquitoes. Hazard Ratio (HR) values, indicating the average daily risk of dying between two groups, were computed to measure significant differences in overall mortality rates. To justify the proportional hazard assumption, plots of survivor functions were used to check Hazard Ratio proportionality.

Permethrin-induced mortality was computed from mosquito mortality rates 24 hrs after permethrin exposure that were corrected for corresponding control mortalities (exposed to blank papers) exceeding the 5% level using the Abbott's formula [34]. For all exposure assays, differences in group means were analyzed for each mosquito population separately, using mortality proportions that were arcsine √ transformed prior to analysis, and compared using a one-way ANOVA (SPSS 16.0) and a Tukey post-hoc test. Comparisons between the different exposure groups (insecticide, fungus or both) used a two-way ANOVA (SPSS 16.0).

Synergy between the two species of fungus and permethrin was analysed by comparing mortality rates induced by combinations of both agents (observed) with the sum of mortalities induced by each agent separately (expected). The expected mortality was calculated using the formula M_e_ = M_f_ + M_i_ (1 - M_f_/100), where M_f_ and M_i_ were the observed percent mortalities caused by the fungus and the insecticide alone [36]. For all fungus-insecticide combinations, these calculated expected mortality percentages were compared with their corresponding observed mortality percentages (M_fi_) using a Paired Samples T-Test in SPSS 16.0, which allowed for pair-wise comparisons between each of the replicate measurements and to exclude potential replicate variations such as differences between mosquito rearing batches, fungus applications and insecticide paper efficacy. Positive M_fi_-M_e_ values were considered synergistic [37]. A significance level of *<*0.05 was used in all analyses.

## Results

### Baseline fungal susceptibility

**Figure 1:**
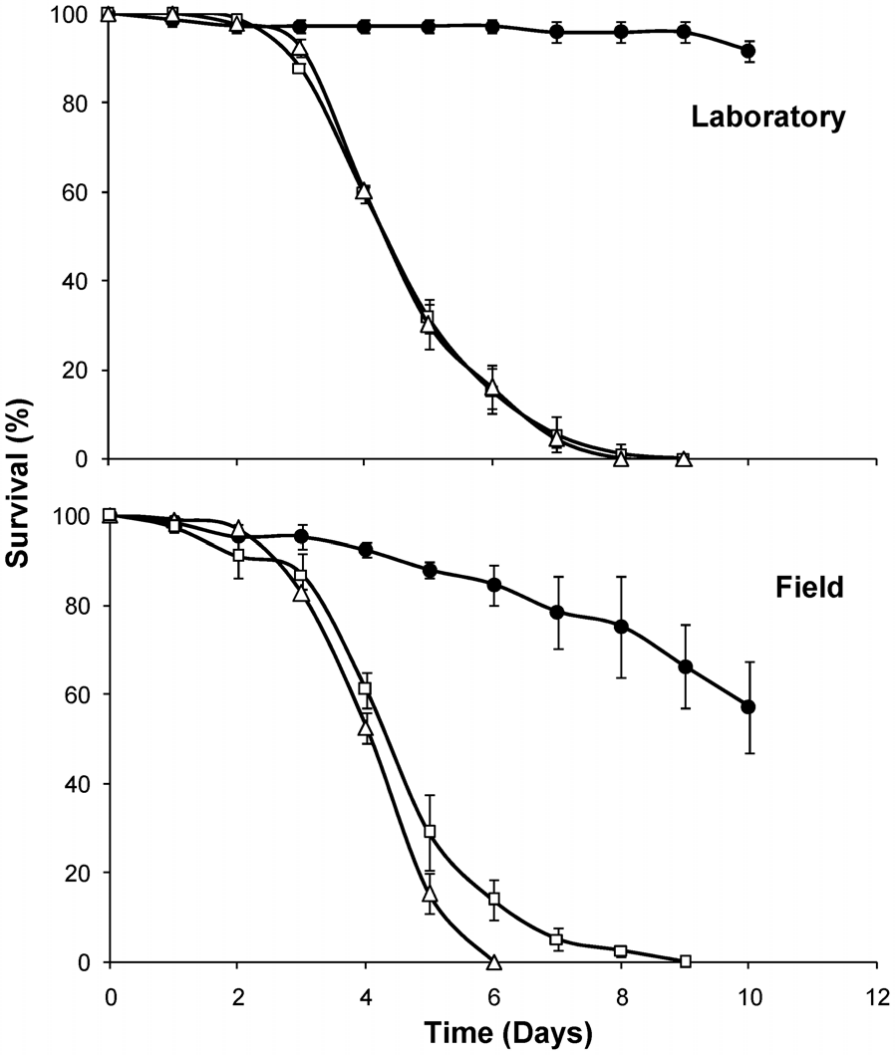
Effect of fungal infection on mosquito survival. Mean (±SEM) cumulative proportional survival of *B. bassiana*-infected (triangles), *M. anisopliae*-infected (squares) and uninfected control mosquitoes (black circles), of the laboratory-reared (top) and field-collected (bottom) insecticide-resistant *An. gambiae s.s.* mosquitoes. Data represent four and three replicates, respectively, of approximately 30 females.

Both laboratory-reared and field-collected insecticide-resistant *An. gambiae s.s*. were susceptible to *M. anisopliae* and *B. bassiana*, with 100% mortality reached within nine days after exposure (Figure 1) and >70% sporulation of cadavers (controls showing 0% sporulation). Survival analysis showed no significant differences in virulence between *B. bassiana* and *M. anisopliae* in the laboratory colony (HR = 1.29, P = 0.09) or the field-collected mosquitoes (HR = 1.35, P = 0.07). There was no significant interaction between fungus treatment and mosquito colony (HR = 0.83, P = 0.16), indicating that fungal infection had a similar impact on *kdr* mosquito longevity in the laboratory and field populations.

### Baseline permethrin resistance

**Figure 2:**
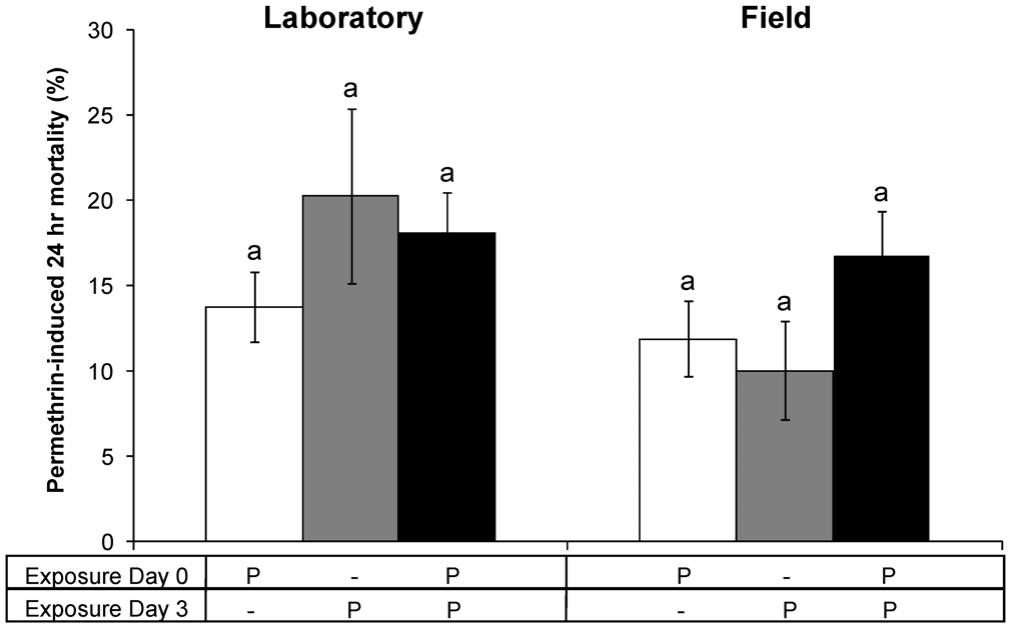
Permethrin sensitivity. Mean (± SEM) proportional mortality of uninfected insecticide-resistant mosquitoes from the laboratory (left) and field population (right) 24 hrs after permethrin-exposure. White bars represent permethrin-induced mortality of 3-day old mosquitoes exposed once on day 0. Grey bars show mortality of 6-day old mosquitoes exposed once on day 3. Black bars show permethrin-induced mortality after a second exposure on day 3 of 6-day old mosquitoes that had survived a first exposure on day 0. From left to right, data depict 10, 5, 5, 8, 4, and 4 replicate groups of 28 females, with significant differences in group means indicated by non-corresponding letters.

Permethrin-induced mortality rates were compared between groups that were exposed to permethrin on day 0 (Group 3), on day 3 (Group 2), or on day 0 + day 3 (Group 4) (Table 1). Control mortalities (unexposed groups) were below 5% and were, therefore, not used to correct the insecticide-induced mortality rates. Both the laboratory VKPer colony and the colony collected in the field were highly resistant to permethrin, exhibiting only 10–20% mortality following single or repeat exposures (Figure 2). Equivalent single insecticide exposure of the susceptible mosquito strains resulted in 100% mortality. Statistical analyses on the group means showed that there were no significant differences in sensitivity to permethrin between the laboratory and field mosquitoes (Figure 2). Moreover, permethrin resistance levels did not increase in the three day test period and were not significantly affected by repeat exposure (Figure 2).

### Effects of fungus-insecticide combinations

**Figure 3:**
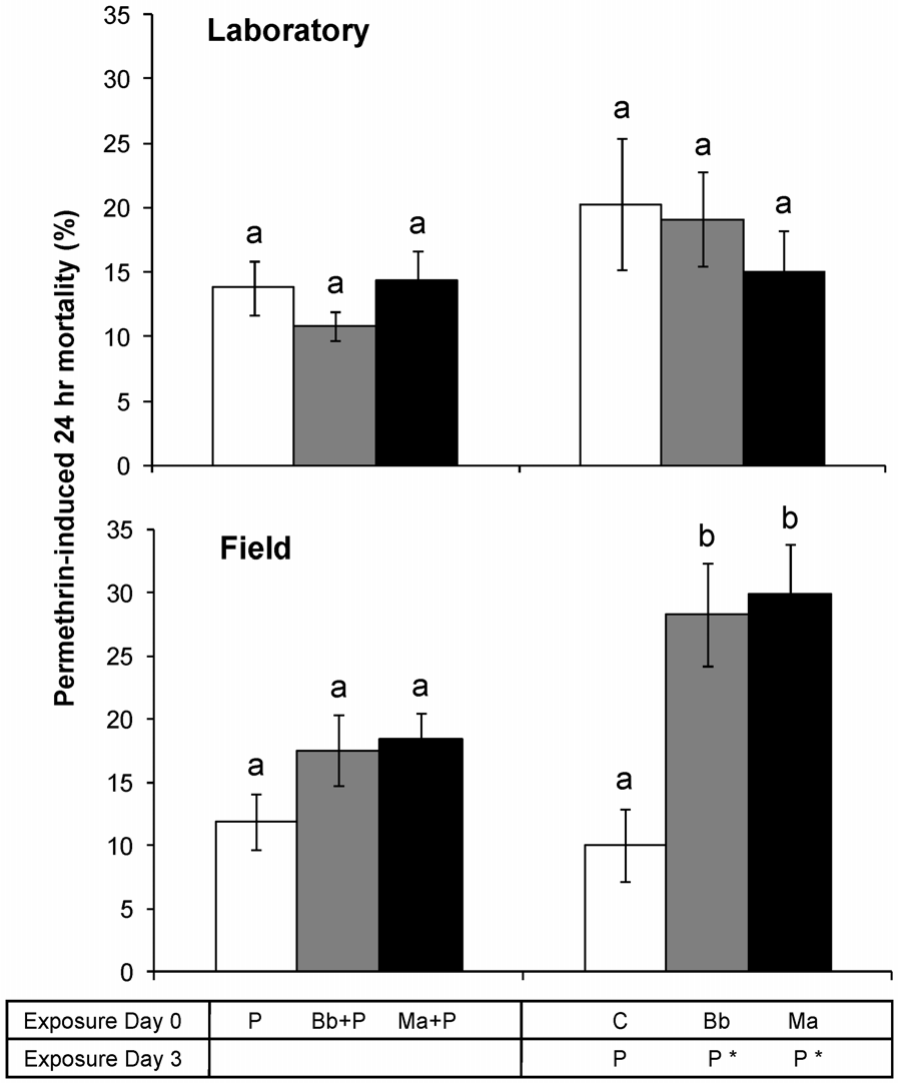
Effect of fungus on permethrin sensitivity. Mean (± SEM) percentage permethrin-induced mortality of uninfected (white), *Beauveria*-infected (grey) and *Metarhizium*-infected (black) mosquitoes from the laboratory colony (top) and field *An. gambiae s.s* population (bottom). Data show mortality rates measured 24 hrs after permethrin exposure on day 0 (left) and day 3 (right), from ten laboratory and eight field replicates of 28 females per group. Mortality rates of fungus-infected groups exposed to permethrin on day 3 (*) were corrected for mortality of corresponding fungus-infected groups exposed to control papers. Significant differences are indicated by non-corresponding letters.

To determine the effect of fungal infection on permethrin efficacy, mortality following permethrin exposure was compared between *Beauveria*-infected, *Metarhizium*-infected and equivalent uninfected groups. Mortality rates of fungus-infected groups exposed to permethrin on day 3 (Groups 6 & 10) were corrected for mortalities of corresponding (fungus-infected) control groups (Groups 5 & 9), whereas for the other treatments no corrections were made since their control mortalities did not exceed the 5% level [34]. Permethrin-induced mortality measured on day 1 was not higher in groups co-exposed to fungus compared with groups exposed to only permethrin (Figure 3) in either mosquito colony, indicating no interactions at the very early stages of fungal infection. However, once fungal infection had proliferated for three days, exposure to permethrin induced significantly higher mortality in the *Beauveria*-infected (Group 6; P = 0.02) and *Metarhizium*-infected (Group 10; P = 0.009) mosquitoes from the field population (Figure 3). These differences in permethrin-induced mortality were not observed in the *kdr* VKPer laboratory colony (Figure 3) even though fungus-induced mortality rates used to correct the co-exposed group mortalities were similar for both colonies.

**Figure 4:**
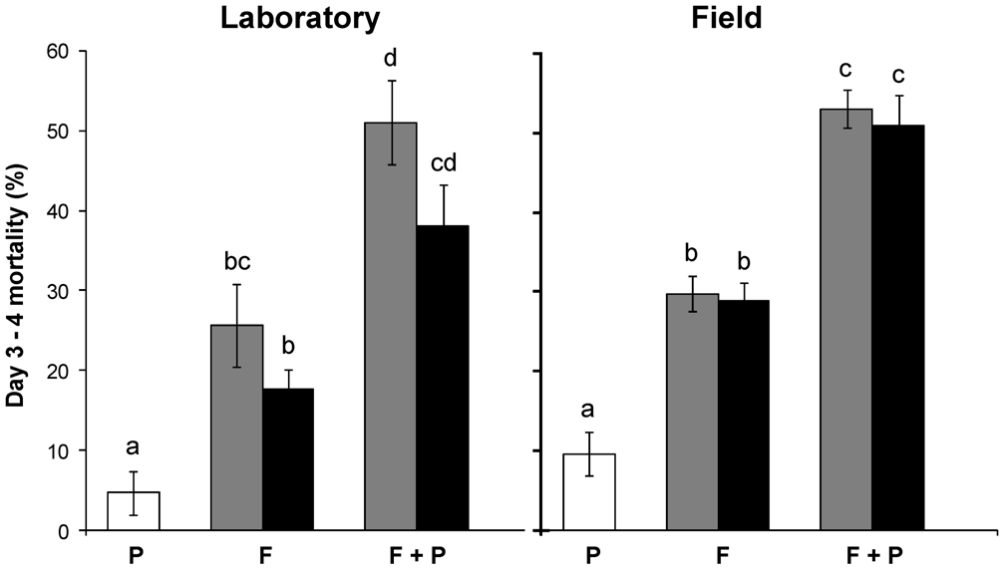
Effect of permethrin on fungal virulence. Mean (± SEM) percentage mortality measured at day 4 of uninfected (white), *Beauveria*-infected (grey) and *Metarhizium*-infected (black) *kdr* mosquitoes from the laboratory colony (left) and field population (right), which were exposed to permethrin (P), fungus (F) or both (F+P) on day 0. Data represent five and four replicates of 28 laboratory and field mosquitoes, respectively. Significant group differences are indicated by non-corresponding letters.

Reciprocal effects of insecticide exposure on subsequent fungal efficacy were assessed by comparing uncorrected mortality rates between day 3 and day 4 for mosquito groups exposed on day 0 to insecticide (Group 3), fungus (Groups 5 & 9), or both (Groups 7 & 11). Exposure to permethrin alone (P) showed minimal impact on mortality rates among mosquito survivors three days later (Figure 4). Consistent with mortality trajectories in Figure 1, exposure to fungus alone (F) resulted in a significantly greater day 3-4 mortality rate compared with uninfected controls (Figure 4). Fungus-induced mortality rates were significantly higher in the fungus and insecticide co-exposure treatments (F+P) (Figure 4), indicating that permethrin augmented the proliferation of *B. bassiana* and *M. anisopliae* in both the laboratory colony and field-collected mosquitoes. All co-exposure treatments were found to interact synergistically, such that day 3-4 mortality rates were significantly higher (P<0.05) than expected from the single treatment effects combined. Further effects on daily mortality rates at the time when wild mosquitoes would be expected to take a second blood meal (e.g. on day 7) could not be analyzed as mortality of mosquitoes exposed to the various fungus-permethrin combination treatments was 80–90% by day 7, and not suitable for comparing synergistic effects of different exposures.

**Figure 5:**
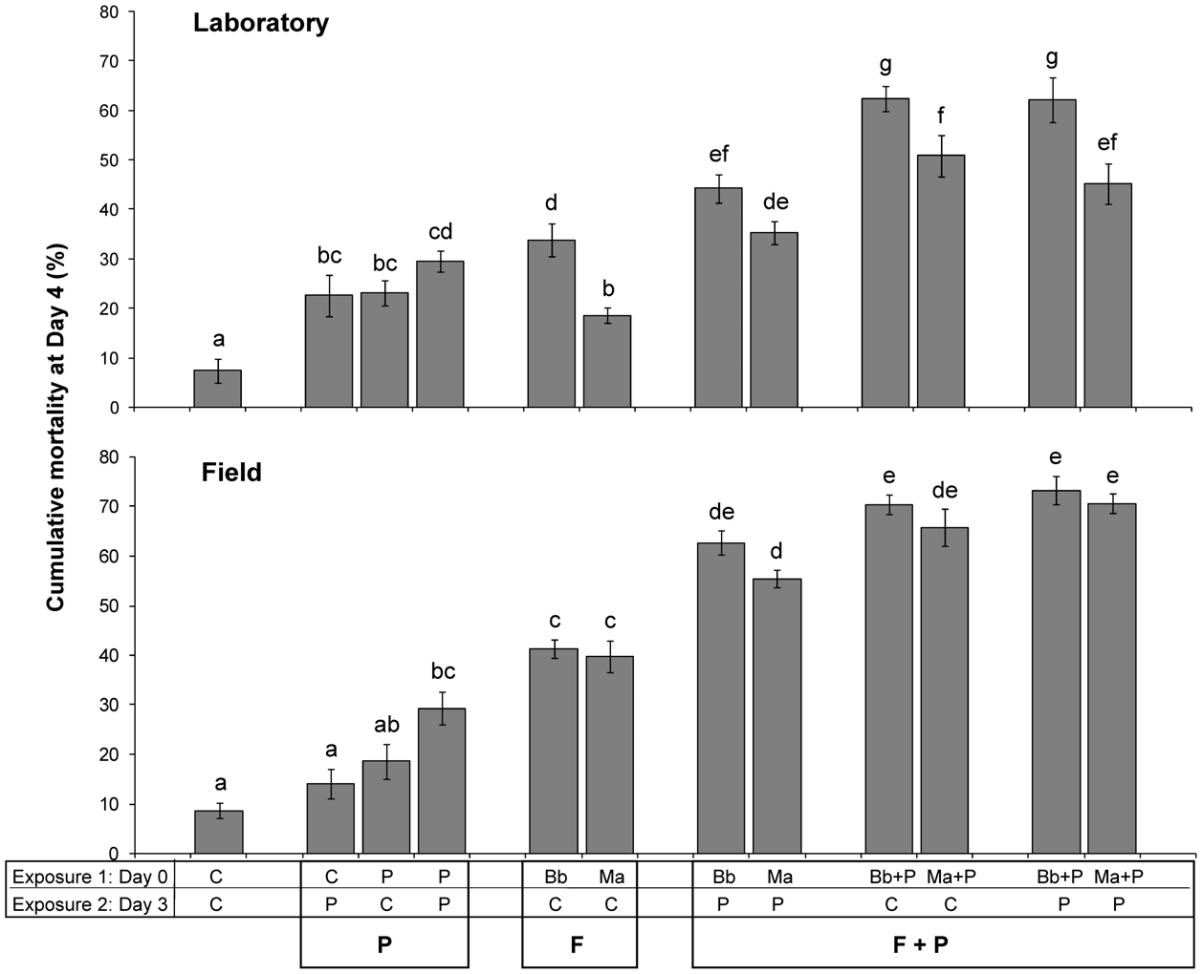
Impact of co-exposure on *kdr* mosquito survival. Efficacy of fungus-insecticide combinations against laboratory-reared VKPer (top) and field-collected *Anopheles gambiae s.s.* from Ladji, Benin (bottom). Mosquitoes were treated with permethrin (P), fungus (F) or combinations of both (F+P) in two subsequent rounds on day 0 and day 3, by exposing them to control papers (C), permethrin papers (P), *B. bassiana*-coated (Bb) or *M. anisopliae*-coated papers (Ma) as indicated on the X-axis. Data represent cumulative proportional mortality (mean ± SEM) measured at day 4, from five and four replicates of 28 laboratory and field mosquitoes, respectively. Significant group differences (separate for both populations) are indicated by non-corresponding letters.

Overall effects of fungus-insecticide combinations were analyzed using uncorrected cumulative day 4 mortality rates, highlighting the total impact within the timeframe of 1–2 mosquito gonotrophic cycles. In the laboratory colony, a single permethrin exposure caused a significant increase in mortality relative to controls, although this was not increased further by a second exposure (Figure 5). In the field population, only the double permethrin exposure was significantly different from the controls. Overall, maximum mortality induced by permethrin was approximately 20–30% compared with 10% in the controls (Figure 5), indicating that permethrin did not have a substantial effect on mortality in these *kdr An. gambiae s.s.* mosquitoes. Effects of fungal infection four days after exposure, though still moderate, were slightly higher, inducing 19–41% mortality (Figure 5). Impact of fungus tended to be marginally higher in the field population, with no marked differences in the effects of *B. bassiana* and *M. anisopliae*. In the laboratory colony, the effect of *B. bassiana* on mosquito survival was greater than *M. anisopliae* in most treatments, with significant differences (P<0.05) indicated by non-corresponding letters in Figure 5.

Both fungus species had a higher impact on mosquito mortality when combined with permethrin. All tested fungus and permethrin combinations (F+P) resulted in higher cumulative mortality compared with the use of permethrin- (P) (P<0.001) or fungus-only (F) (P<0.001) treatments in both mosquito strains (Figure 5). Co-exposure to both agents on day 0 induced highest overall mortality (in the order of 60–70%), with no additional mortality from a second exposure to permethrin (Figure 5).

In the field population, simultaneous co-exposure to *B. bassiana* or *M. anisopliae* and permethrin (Groups 7,8,11,12), as well as sequential exposure to *B. bassiana* and then permethrin (Group 6), induced significant synergistic increases in the cumulative mortality at day 4 (Table 2). In the laboratory mosquito colony, significant synergy between fungus and permethrin was observed only in the single co-exposure treatments (Groups 7 & 11) (Table 2).

**Table 2:** Synergistic interactions between fungus and permethrin.

Exposure		Laboratory				Field			
Day 0	Day 3	Observed	Expected*	T-test	P	Observed	Expected*	T-test	P
Bb	Perm	44.3±2.9	49.0±3.2	1.13	0.323	62.7±2.4	49.8±0.6	4.34	**0.023**
Bb+Perm	Control	62.4±2.5	49.3±2.6	4.63	**0.010**	70.3±2.0	52.5±1.1	9.81	**0.002**
Bb+Perm	Perm	62.2±4.5	56.2±3.2	1.44	0.223	73.2±2.9	58.6±1.5	9.95	**0.009**
Ma	Perm	35.3±2.3	37.1±3.5	0.38	0.726	55.4±1.7	49.1±3.7	1.67	0.194
Ma+Perm	Control	50.9±4.2	37.5±2.1	3.46	**0.026**	65.8±3.7	50.7±4.5	11.4	**0.001**
Ma+Perm	Perm	45.2±4.2	42.6±2.1	0.66	0.547	70,6±2.0	57.3±3.4	3.84	**0.031**

*Expected mortality (M_e_)  =  M_f_ + M_i_ (1 - M_f_/100), with M_f_ and M_i_ being observed percent mortalities caused by the fungus and the insecticide alone respectively.

Synergistic effects between permethrin (Perm) and the fungus *Beauveria* (Bb) or *Metarhizium* (Ma) on laboratory (df = 4) and field (df = 3) *kdr* mosquito survival. Results show outcomes of paired-samples T-test comparisons of observed and expected cumulative day 4 mortality rates (mean ± SE), with significant synergy indicated in bold.

## Discussion

The laboratory colony (VKPer) and field population of *An. gambiae s.s* from West Africa showed limited sensitivity to permethrin following single or multiple exposures across the duration of a gonotrophic cycle. These results are consistent with known high levels of *kdr* expression in these populations. While size or other fitness parameters (not measured) may be expected to be more variable in the adults reared from field-collected larvae and pupae, their baseline insecticide sensitivity was similar to laboratory-reared mosquitoes and was consistent between the different experiments.

Both populations of *kdr* mosquitoes were highly susceptible to two candidate isolates of *B. bassiana* and *M. anisopliae*. Exposure to an intermediate dose of fungus using a standard WHO bioassay caused 100% mortality within nine days. This treatment mortality was substantial higher than the control mortality, even in the Cotonou laboratory where survival rates of the field-collected mosquitoes were slightly reduced. Sporulation of fungal cadavers tended to be lower in the *Metarhizium*-infected field mosquitoes, which is consistent with findings that this fungus is not a strong competitor of other microbiota and that hyphal growth can be affected by environmental factors [33]. Mortality data, however, indicated high fungal infectivity of both isolates in both mosquito populations. These observations confirm findings from recent studies on the same [30] and other resistant mosquito species and strains [18], [29], and demonstrate for the first time that also wild populations of West African pyrethroid-resistant *An. gambiae s.s.* do not confer resistance to insect-pathogenic fungi. Given the growing problems of pyrethroid resistance and issues of cross-resistance to DDT among malaria vectors, these results highlight an important strength of the bio-insecticidal approach.

Impact on survival was broadly similar for both isolates, although some of the test results suggest slightly reduced efficacy of *M. anisopliae*, which is likely linked to a lower quality of the production batch available for those tests, which showed lower viability on agar than the *B. bassiana* spores (70% vs 92%). Other findings, however, also indicate a higher persistence of *Beauveria* spores [26], which implies that this fungus may be more suitable for field implementation. Spore virulence and persistence can differ greatly between different fungal strains within and between hyphomycetous species, and can be optimized through production methods and formulation [33]. Ultimately, the choice of fungal strain will require evaluations of the long-term effectiveness of different species and isolates after application under realistic field conditions, together with evaluation of other operational criteria such as mass production efficiency, long-term storage viability and (eco)toxicology [38].

Beyond the ability to infect insecticide-resistant mosquitoes, this study identified the potential for synergistic interactions between fungi and pyrethroids. Firstly, pre-infection with fungus led to an increase in permethrin-induced mortality levels, i.e. the ‘instantaneous’ mortality resulting from exposure to permethrin. This effect was restricted to the field mosquito population and was not apparent in the laboratory colony. The mechanism for this effect is unclear. Previous work suggested that fungal metabolites may interfere with enzymatic insecticide resistance mechanisms [18] and so it is possible that the observed effects in *An. gambiae* from Ladji result from an effective increase in sensitivity to permethrin in the presence of a proliferating fungal infection. While both *An. gambiae* populations are known to express *kdr* and such effects would not necessarily be expected where resistance is conferred by target-site insensitivity alone, the VKPer laboratory colony has been fixed for *kdr* resistance through repeated selection and maintained in the laboratory for many years [7], whereas elevated levels of oxidases and esterases have been reported for the wild *An. gambiae s.s.* population at Ladji, Benin [2]. Thus, the differences in response to permethrin between fungus-infected laboratory and field-collected mosquitoes could be indicative of more complex multiple resistance mechanisms operating in the field. The slightly more variable environmental conditions in the Cotonou laboratory might, however, also have affected fungal efficacy and survival rates of field-collected mosquitoes.

Secondly, simultaneous exposure to fungus and permethrin increased the daily mortality rate of mosquitoes at the point where fungus starts to proliferate within the insect and approaches its exponential growth phase [see [20]]. This higher fungal virulence three days post-exposure is most likely caused by indirect effects of the insecticide, since pyrethroids are usually rapidly detoxified by metabolization processes [39] and so would no longer be present inside the insect body at that time-point. Although the exact mechanisms for this effect are unclear, insecticides may affect the insect cuticle and facilitate fungal penetration, or may inhibit cellular and humoral immune responses and facilitate fungal infection inside the body as shown in other insect species [40], [41].

Finally, in several combination treatments, and particularly simultaneous exposures, synergistic interactions between fungus and permethrin on overall mosquito mortality were observed. These synergistic effects resulted in approximately 50–70% mortality after four days in most co-exposed groups, compared with 15–40% for permethrin or fungus alone. There was no additional mortality in co-exposed groups after a repeat exposure to insecticide, which suggests that effects of insecticide on fungal proliferation contribute significantly to the overall impact and that a single insecticide exposure at the start is sufficient to induce synergy.

Several theoretical studies have demonstrated that the relatively slow speed of kill of entomopathogenic fungi can be sufficient to impact on malaria transmission since the extrinsic incubation period of the malaria parasite within the mosquito (typically 10–14 days in high transmission settings) creates a window of several days for the fungus to act [19], [31], [42], [43]. There may even be evolutionary benefits in slow speed of kill [38], [43]. However, for a slow-acting product to be effective, coverage needs to be sufficiently high to ensure contact with mosquitoes early in adult life, otherwise they might escape the negative effects of fungal infection long enough to transmit malaria [31]. Accordingly, the synergistic effects of fungus and permethrin on mortality could be very important; 50–70% mortality within four days has the potential to dramatically reduce malaria transmission across the duration of 1–2 gonotrophic cycles and could enable control at more moderate levels of coverage. More fundamentally, adding fungal entomopathogens could make malaria control possible where insecticide resistance has rendered pyrethroids essentially ineffective.

Operational deployment of fungal bio-insecticides for mosquito control requires further research and development, including development of feasible field delivery methods that are compatible with the current chemical controls tools already in place [38], [44]. In the current study, all fungus-insecticide combinations had a significantly higher impact on mosquito survival than fungus or insecticide alone. However, given that co-exposure produced the strongest synergistic effects, it would be interesting to explore delivery systems that promote more or less simultaneous exposure to both products during a single feeding episode, such as using LLINs together with indoor residual fungal treatments or fungus-treated resting targets [16], [19] that can be visited shortly before or after contact with a bednet, or combining fungi and (non-repellent) insecticides on single substrates such as walls, bednets or eave curtains (results from other studies show good compatibility of fungus-insecticide mixtures [45]–[47]). Moreover, although consistent with standard WHO methods, the type of exposure assays used in the current laboratory study do not directly simulate fungal exposure as might be expected to occur in the field. Further research is, therefore, required to determine the effects of more realistic fungal exposures (e.g. transient contact from resting on different substrates) and their robustness across different environmental conditions. Equally important would be to explore dose-dependent effects and test whether fungal infection can enhance the efficacy of sublethal insecticide doses, which has been shown to be the case in other insect species [41], [48], [49].

Currently there is great interest in using combination interventions with distinct modes of action as management strategy, not only to control resistant mosquitoes but to delay the selection of novel resistance, which indicates a potential role for fungi with other categories of insecticide. Such research could enable the development of novel integrated vector management (IVM) strategies that would sustain the useful lifespan of current insecticide-based interventions and maximize control in the face of emerging insecticide resistance.
